# Seasonal Changes in *Pinus tabuliformis* Root-Associated Fungal Microbiota Drive N and P Cycling in Terrestrial Ecosystem

**DOI:** 10.3389/fmicb.2020.526898

**Published:** 2021-01-18

**Authors:** Hai-Hua Wang, Hong-Long Chu, Qing Dou, Huan Feng, Ming Tang, Shuo-Xin Zhang, Chun-Yan Wang

**Affiliations:** ^1^College of Forestry, Northwest A&F University, Yangling, China; ^2^Department of Food Science and Technology, College of Agriculture and Biotechnology, Chungnam National University, Daejeon, South Korea; ^3^College of Biological Resource and Food Engineering, Center for Yunnan Plateau Biological Resources Protection and Utilization, Qujing Normal University, Qujing, China; ^4^College of Forestry and Landscape Architecture, South China Agricultural University, Guangzhou, China; ^5^Qinling National Forest Ecosystem Research Station, Huoditang, Ningshan, China

**Keywords:** temperate forest, Pinus tabuliformis, root-associated fungi, seasons, microbial network, soil function

## Abstract

In terrestrial ecosystems, mycorrhizal roots play a key role in the cycling of soil carbon (C) and other nutrients. The impact of environmental factors on the mycorrhizal fungal community has been well studied; however, the seasonal variations in the root-associated fungal microbiota affected by environmental changes are less clear. To improve the understanding of how environmental factors shape the fungal microbiota in mycorrhizal roots, seasonal changes in *Pinus tabuliformis* root-associated fungi were investigated. In the present study, the seasonal dynamics of edaphic properties, soil enzymatic activities, root fungal colonization rates, and root-associated fungal microbiota in *P. tabuliformis* forests were studied across four seasons during a whole year to reveal their correlations with environmental changes. The results indicate that the soil functions, such as the enzymatic activities related to nitrogen (N) and phosphorus (P) degradation, were varied with the seasonal changes in microclimate factors, resulting in a significant fluctuation of edaphic properties. In addition, the ectomycorrhizal fungal colonization rate in the host pine tree roots increased during warm seasons (summer and autumn), while the fungal colonization rate of dark septate endophyte was declined. Moreover, the present study indicates that the fungal biomass increased in both the pine roots and rhizospheric soils during warm seasons, while the fungal species richness and diversity decreased. While the Basidiomycota and Ascomycota were the two dominant phyla in both root and soil fungal communities, the higher relative abundance of Basidiomycota taxa presented in warm seasons. In addition, the fungal microbial network complexity declined under the higher temperature and humidity conditions. The present study illustrates that the varieties in connectivity between the microbial networks and in functional taxa of root-associated fungal microbiota significantly influence the soil ecosystem functions, especially the N and P cycling.

## Introduction

In terrestrial ecosystems, soil microorganisms account for 85–90% of soil ecosystem functional processes (Djukic et al., 2013). Soil organic matter (SOM) enters the soil in the form of plant litter (Berg and Smalla, 2009; Rodeghiero et al., 2010), and its degradation depends mainly on three interacting factors: the chemical composition of the litter, soil physical properties, and biotic factors (Polyakova and Billor, 2007). Previous studies have shown that plant litter is initially degraded by soil rot fungi, as these fungi can produce a wide range of extracellular enzymes that target the recalcitrant lignocellulose matrix, which is the most abundant component of plant biomass and cannot be decomposed by other organisms (Hammel, 1997; Boer et al., 2005; Steffen et al., 2007; Baldrian et al., 2011; Schneider et al., 2012; Wang et al., 2018). Therefore, as one of the major components of the soil community, the fungal community is crucial for ecological recycling. However, less research has been conducted on soil fungi than on soil bacteria.

In general, the soil fungal community is affected by aboveground plants (Djukic et al., 2010; A’Bear et al., 2012); soil depth; and other soil factors, such as soil moisture, temperature, pH, organic matter, and the carbon (C)/nitrogen (N) ratio (Waldrop et al., 2006; Voříšková et al., 2014; Wang et al., 2015). Biotic factors, such as plant growth and vegetation composition, are considered to drive changes in root-associated fungal communities over time (Dumbrell et al., 2011) because of the provisioning of C or root exudation (Brzostek and Finzi, 2011; Drake et al., 2011). In addition, edaphic factors play a crucial role in the distribution of these fungal communities (DeBellis et al., 2006).

The plants form various interactions with microbiota ranging from mutualism to parasitism, which are pivotal to plant performance. Root-associated fungi play important roles in plant health and growth *via* acquisition of modulating plant nutrient acquisition, root architecture, and tolerance to abiotic stress (Schachtman et al., 1998). Mycorrhizal fungi can form symbioses with approximately half of the tree species present in the temperate forest biome (Phillips et al., 2013), transporting C secreted from roots deep in the soil through mycelia (Lindahl et al., 2007; Voříšková et al., 2014). The plants receive phosphorus (P) and other nutritional elements from these fungi in exchange for photosynthates (Hobbie, 2006; Van Der Heijden and Horton, 2009). Several studies have shown that ectomycorrhizal (ECM) roots produce a suite of extracellular enzymes which enter the soil (Talbot et al., 2008; Puissant et al., 2015). For instance, some of these ECM fungi produce enzymes that hydrolyze organic forms of P and nitrogen N (Häussling and Marschner, 1989; Joner et al., 2000; Read and Perez Moreno, 2003). Dark septate endophytes (DSEs) in plant roots can live under extreme conditions, such as under heavy metal contamination or drought stress and in saline-alkali or geothermal soils, because of the presence of melanin in their hyphae (Redman et al., 2002; Postma et al., 2007; Ban et al., 2012).

Host plants are expected to be among the most important factors driving the response of root-associated fungal communities to ecosystem development during seasons (Dickie et al., 2013; Fukami and Nakajima, 2013; Jangid et al., 2013). For instance, different types of mycorrhizal fungal communities are generally confined to specific plant groups, and some mycorrhizal fungal species form symbioses with a narrow range of plant species (Smith and Read, 2007). In a research on primary succession of on dunes, edaphic properties strongly determined the growth patterns of mycorrhizal fungi (Sikes et al., 2014). In studies addressing the fertilization of agricultural soils, Verbruggen et al. (2012) and Gosling et al. (2013) reported that the richness and biomass of root-associated fungal communities decreased when the concentration of soil nutrients increased. In addition, edaphic properties, such as pH, P, N, potassium (K), calcium (Ca), and temperature, have also been reported as driving factors that influence the structure and function of root-associated fungal communities (Högberg et al., 2010; Põlme et al., 2013).

The underground fungal community is well-documented to be one of the most sensitive bioindicators of environmental change, and many studies have investigated the responses of this community to changes in edaphic properties (van Der Linde et al., 2018). However, much uncertainty concerning the effects of seasonal changes in root-associated fungal microbiota on soil ecosystem functions remains. Thus, studies on the seasonal dynamics of root-associated fungal communities are necessary to gain insights into the correlations between soil ecosystem functional variations and fungal microbiota. In the present study, the correlations between soil ecosystem functional processes and root-associated fungal communities in temperate forest ecosystems are expected to be revealed across seasons. *Pinus tabuliformis* is one of the dominant plant species in temperate forests in China, and as a typical ECM plant, this forests play a key role in soil ecosystem cycling. In this study, the seasonal dynamics of root-associated communities of *P. tabuliformis* in temperate forests in the Qinling Mountains were studied. Moreover, seasonal changes in edaphic properties, soil enzymatic activities, and root-associated fungal colonization rates were also measured and analyzed.

## Materials and Methods

### Experimental Site and Sampling

This study was conducted at the Huoditang Forest Research Station (latitude 33°18'-33°28'N, longitude 108°21'-108°39'E) in Ningshan County, Ankang city, Shaanxi Province; the station is located on a south-facing slope of the Qinling Mountains. The altitude of this region ranges from 800 to 2,500 m above sea level (asl), and four distinct seasons occur. The annual mean temperature is 8–10°C, the mean precipitation is 900–1,200 mm, the mean evaporation is 800–950 mm, the average humidity is 77%, and the frost-free period lasts 170 days. Most of the rainfall occurs from July to September, snowfall begins in early October, and thawing occurs early the following May (China Meteorological Data Service Center). The soil is a mountain brown earth, classified by the Eutric Regosol in the Food and Agriculture Organization (FAO) of the United Nations system, with a depth ranging from 30 to 50 cm. Intensive selective logging that occurred during the 1960s and 1970s contributed to the regeneration of diverse natural secondary forests (NSFs). Many *P. tabuliformis* forests are distributed from 1,300 to 2,300 m above sea level, and all of them are of the same age. The aforementioned factors all indicate that such forests are an optimal environment to investigate the temporal distributional patterns and seasonal variations in the underground fungal communities associated with ECM plants.

Sampling in *P. tabuliformis* forests was performed throughout the year during summer (16 August 2014), autumn (3 November 2014), winter (20 January 2015), and spring (8 May 2015). According to seasonal variations, the seasons were classified as warm seasons (summer and autumn) and cold seasons (winter and spring). The newly fallen plant litter was considered to indirectly influence the underground fungal community due to the thick layer of litter accumulation on the ground. To reduce differences caused by other factors, three *P. tabuliformis* forests of similar age and stand slope (with minor variations in their forest stand and grass structure) at the same altitude (1,900 m asl) were selected as sampling sites. At each site, five *P. tabuliformis* individuals that were at least 10 m apart were randomly selected and sampled. All samples were collected at a distance of 1.5 m from the base of the pine tree and were approximately equidistant from one another. After removing the litter from the forest floor, three soil cores were collected from the soil layer (0–30 cm) using a hand-held power sampler. The three cores from near the same tree were pooled together to form a single composite sample for the subsequent analysis of edaphic properties. Moreover, the roots of *P. tabuliformis* were also sampled at the same locations as the cores. The roots were traced to the originating tree to ensure their identity, and loosely adhered soil was gently shaken off. And then rhizosphere soil, tightly associated with roots, was collected by brushing the soils. The soil and root samples were collected from the same locations each season. Therefore, a total of 60 soil samples and 60 root samples were collected and taken to the laboratory for subsequent analyses.

The root samples were washed with tap water in a 1 mm sieve to remove the soil and organic particles. Afterward, root tips (approximately 2,000 per sample) were randomly picked from each root sample and placed in a 2 ml tube after they were rinsed three times with sterile water (Chu et al., 2016). Both the root tip samples and rhizospheric soil samples were stored in a freezer at −80°C before DNA extraction. The remaining fine roots were collected and stored at 4°C for fungal colonization detection. The soil samples were divided into two parts after sieving with a 1 mm sieve: one part was stored in a freezer at −80°C for DNA extraction, and the other part was air-dried for edaphic property measurements.

### Measurements of Edaphic Properties

To reveal the soil condition, the microclimate factors and the nutrient content were measured. Soil temperature (ST) was measured during the sampling process using an angle-stem earth thermometer embedded into the soil at depths of 5, 10, 15, 20, and 25 cm; three points at each sampling location were measured, after which a mean value was calculated. The soil moisture content (SMC) was analyzed using the method described by the Association of Official Analytical Chemists (AOAC; Chemists and Horwitz, 1990). The soil total nitrogen (TN), total phosphate (TP), and total potassium (TK) contents were measured by inductively coupled plasma-atomic emission spectrometry (ICP-AES) after digestion with HNO_3_ + HClO_4_. The ammonium-nitrogen (NH_4_-N) and nitrate-nitrogen (NO_3_-N) in the soils were extracted with KCl, after which their contents were measured with a SEAL AutoAnalyzer 3 instrument (AA3, Germany). The soil available potassium (AK) and available phosphorus (AP) contents were measured by ICP-AES after extraction with NH_4_OAc and NH_4_HCO_3_ + diethylenetriamine-pentaacetic acid (DTPA), respectively. The soil pH was determined according to the potentiometric method (Tan, 2005), and the SOM content was measured using the potassium dichromate volumetric method (Nelson and Sommers, 1996).

Soil catalase and urease activities were measured using the KMnO_4_ titrimetric method (Tabatabai, 1994) and colorimetric determination of ammonium (Kandeler and Gerber, 1988), respectively. Soil acid phosphatase and dehydrogenase activities were determined by the methods reported by Tabatabai (1994), and soil sucrase activity was measured as described by Guan et al. (1986).

### Measurements of Root Fungal Colonization Rates

The *P. tabuliformis* fine root segment samples were washed several times with tap water and cleared in 10% KOH (w/v) at 90°C for 7–8 h depending on the degree of root pigmentation. The cooled root samples were subsequently dyed, and the colonization rates were measured with the method described by Chu et al. (2016). The ECM fungal hyphae were dyed blue and distinguished from other fungi by a Hartig net and a fungal sheath; moreover, the DSE hyphae could not be stained and were dark red-brown to dark brown. The fungal colonization rates were ultimately measured under a compound light microscope at 10 × 20 magnification using the gridline intersect method (Giovannetti and Mosse, 1980), and 500 intersections were evaluated for each root sample. The number of intersections with ECM, DSE hyphae, or microsclerotium was recorded. The colonization rate was calculated as follows: (number of intersections with fungal colonization)/(total number of counted interactions) * 100%.

### DNA Extraction, PCR, and Sequencing

A total of 60 root samples and 60 rhizospheric soils were used for DNA extraction. Genomic DNA was extracted from 0.5 g of homogenized root tips and soils using an HP Plant DNA Kit and a Soil DNA Kit (Omega Biotek, USA), respectively, according to the manufacturer’s instructions and served as the template for PCR amplification. Using the primer pair nu-SSU0817 5'-barcode-TTAGCATGGAATAATRRAATAGGA-3' and nu-SSU1196 5'-TCTGGACCTGGTGAGTTTCC-3', in which “barcode” is an eight-base sequence unique to each sample (Borneman and Hartin, 2000), partial fungal 18S rDNA genes were amplified in a 20 μl reaction mixture comprising 10 ng of template DNA, 4 μl of 5× FastPfu Buffer (Bio-Rad, USA), 2 μl of 2.5 mM dNTPs, 0.4 μl of FastPfu Polymerase (Tiangen Biotech Co., Ltd., Beijing, China), 0.4 μl of each primer (5 μM), and 3.2 μl of ddH_2_O. The amplification was performed on a thermal cycler (Bio-Rad, USA) programmed for the following conditions: 85 s at 94°C; 35 cycles of 35 s at 94°C, 55 s at 55°C, and 45 s at 72°C, with a final extension step for 10 min at 72°C. The PCR products were purified with Agencourt AMPure XP SPRI magnetic beads and then quantified with a Qubit 2.0 Fluorometer (Invitrogen) and a Qubit dsDNA HS Assay Kit (Invitrogen). Five purified PCR products from the same investigated site and season were pooled to obtain a mixed equimolar amplicon library. Finally, 12 mixed soil libraries and 12 mixed root amplicon libraries were developed. After they were normalized, these amplicons were sequenced on an Illumina MiSeq platform (Majorbio, Shanghai, China; Caporaso et al., 2012).

Sequences obtained from the Illumina MiSeq platform were filtered for quality and trimmed to remove the multiplexes and primers using QIIME (version 1.17; Caporaso et al., 2010). The 250-bp reads were truncated at each site with an average quality score <20 over a 10 bp sliding window, and the truncated reads that were shorter than 50 bp were discarded. Moreover, the low-quality reads were removed. Only sequences that overlapped for more than 10 bp were assembled according to their overlapping sequence; otherwise, they were discarded.

The filtered sequences were clustered into operational taxonomic units (OTUs) based on 97% similarity using UPARSE (version 10.0, http://drive5.com/uparse/), and chimeric sequences were identified and removed using UCHIME. The representative sequences of the OTUs were identified with those of reference taxa in the UNITE database (Nilsson et al., 2018), and the lifestyle assignment of these taxa was performed with FUNGuild (Nguyen et al., 2016). The OTUs were used for the subsequent statistical analysis.

### Statistical Analysis

The one-way ANOVA comparisons between groups were performed using SPSS (version 20.0; SPSS, Chicago, IL, USA). Spearman’s correlation analysis was performed to evaluate the relationships between the seasonal changes in edaphic factors, soil enzymatic activities, fungal colonization rates of *P. tabuliformis* roots, the fungal community diversities, and compositions associated with the *P. tabuliformis* roots and rhizospheric soils. The significance of the difference between fungal community compositions was tested by a permutational multivariate analysis of variance (PERMANOVA) of the Bray-Curtis dissimilarity using the vegan package in R.

Alpha diversities of fungal communities were calculated using 20,000 reads per sample with the vegan package (Dixon, 2003). The relative abundances of fungal OTUs were visualized using a nonmetric multidimensional scaling (NMDS) analysis with the vegan package to elucidate the dissimilarities in the fungal community composition across seasons. Furthermore, Venn’s and Sankey’s diagrams were generated using the fungal community OTU numbers in different seasons. The networks were constructed based on the edaphic properties and the selected OTUs (relative abundance >0.1%) using Spearman’s correlation analysis. In addition, the network of each season was performed with OTUs in samples obtained from a single season. Spearman’s correlations were considered to be statistically robust when Spearman’s correlation coefficient (*ρ*) was >0.6 and the value of *p* was <0.01 (Barberán et al., 2012). The topological properties of the networks were calculated, and the co-occurrence networks were visualized in CYTOSCAPE version 3.2.0 (Shannon et al., 2003). To assess the nonrandom pattern in the resultant network, 10,000 Erdös-Rényi random networks were generated for comparison with the topology of the real network using the igraph package.

## Results

### Edaphic Properties and Soil Enzymatic Activities

According to the meteorological data, the highest monthly average temperature (MAT), monthly average precipitation (MAP), and monthly average humidity (MAH) occurred during the summer, followed by autumn and spring (China Meteorological Data Service Center). The edaphic microclimate factors evaluated in this study were significantly affected by climatic changes (Table 1). All of the measured edaphic physicochemical parameters varied significantly among the seasons (*p* < 0.05; Table 1), including ST (*p* < 0.001), SMC (*p* < 0.001), SOM (*p* < 0.05), NH_4_-N (*p* < 0.05), AP (*p* < 0.001), TP (*p* < 0.001), TK (*p* < 0.001), NO_3_-N (*p* < 0.001), TN (*p* < 0.05), AK (*p* < 0.001), and pH (*p* < 0.001).

**Table 1:** Edaphic factors and soil enzymatic activities in *Pinus tabulaeformis* forests located on the Qinling Mountains.

Edaphic factor	Season				*F*	*p*
	Summer (August 2014)	Autumn (November 2014)	Winter (January 2015)	Spring (May 2015)		
Sampling time	August 14, 2014	November 14, 2014	January 15, 2014	May 15, 2015		
MAT (°C)	18.1	7.2	−8.5	3.5		
MAP (mm)	164.6	31.5	6.2	53.7		
MAH (%)	84	82	74	72		
SMC (%)	0.187 ± 0.008^b^	0.283 ± 0.002^a^	0.134 ± 0.002^c^	0.161 ± 0.002^b^	89.024	^***^
ST (°C)	16.72 ± 0.01^a^	8.65 ± 0.03^b^	1.23 ± 0.03^d^	4.72 ± 0.03^c^	23,991	^***^
pH	4.96 ± 0.02^c^	5.2 ± 0.04^b^	4.88 ± 0.07^c^	6.10 ± 0.09^a^	34.47	^***^
TK (g/kg)	3.437 ± 0.20^b^	2.738 ± 0.072^c^	2.642 ± 0.072^c^	4.525 ± 0.003^a^	42.45	^***^
TN (g/kg)	2.19 ± 0.17^b^	2.933 ± 0.001^a,b^	3.342 ± 0.127^a^	2.457 ± 0.015^b^	4.18	^*^
TP (g/kg)	0.22 ± 0.007^c^	0.2 ± 0.006^c^	0.351 ± 0.005^b^	0.579 ± 0.003^a^	25.47	^***^
AP (mg/kg)	10.358 ± 1.03^b^	14.32 ± 0.665^a^	11.381 ± 0.562^b^	5.785 ± 0.169^c^	14.975	^***^
AK (mg/kg)	50.446 ± 1.912^c^	116.728 ± 0.657^a^	117.55 ± 0.609^a^	89.382 ± 4.387^b^	11.39	^***^
NO_3_-N(mg/kg)	3.357 ± 0.182^b^	3.405 ± 0.088^b^	1.791 ± 0.077^c^	9.81 ± 0.885^a^	48.95	^***^
NH_4_-N(mg/kg)	21.839 ± 1.805^a,b^	27.569 ± 1.133^a^	19.866 ± 0.077^b^	17.086 ± 0.3^b^	3.25	^*^
SOM (g/kg)	40.13 ± 5.47^b^	49.526 ± 0.338^a,b^	56.868 ± 4.367^a^	42.214 ± 1.133^a,b^	2.903	^*^
Sucrase activity (mg glucose g^−1^ h^−1^)	0.102 ± 0.01^a^	0.11 ± 0.008^a^	0.106 ± 0.007^a^	0.102 ± 0.007^a^	0.232	N.S.
Urease activity (mg NH_3_-N g^−1^ h^−1^)	4.241 ± 0.299^b^	5.519 ± 0.421^a,b^	6.094 ± 0.605^a^	4.887 ± 0.378^a,b^	3.314	^*^
Dehydrogenase activity (mg Ph(OH) g^−1^ h^−1^)	0.877 ± 0.139^a^	0.884 ± 0.112^a^	0.679 ± 0.099^a^	1.033 ± 0.127^a^	1.44	N.S.
Phosphatase activity (mg Ph(OH)_3_ g^−1^ h^−1^)	0.014 ± 0.001^b^	0.019 ± 0.001^a^	0.015 ± 0.001^b^	0.012 ± 0.001^b^	8.182	^***^
Catalase activity (mg KMnO_4_ g^−1^ h^−1^)	6.726 ± 0.35^b^	8.066 ± 0.111^a^	6.378 ± 0.248^b^	5.329 ± 0.403^c^	14.533	^***^

The data represent the mean ± standard deviation. Different small letters a-d indicate significant differences among different seasons for that parameter. Means were compared by using one-way ANOVA at the *p* < 0.05 level. In this study, the environmental factors of monthly average temperature (MAT), monthly average precipitation (MAP), monthly average humidity (MAH), soil moisture content (SMC), soil temperature (ST), and pH were measured; soil nutrients soil total potassium (TK), total nitrogen (TN), total phosphate (TP), ammonium-nitrogen (NH_4_-N), nitrate-nitrogen (NO_3_-N), available potassium (AK), available phosphorus (AP,) and soil organic matter (SOM) were analyzed; additionally, soil enzymatic activities of sucrase, urease, dehydrogenase, acid phosphatase, and catalase were estimated. Fifteen replicates were used to calculate the mean and standard deviation for each treatment. *Significant at *p* < 0.05; **Significant at *p* < 0.01; ***Significant at *p* < 0.001; N.S, no significant difference. The symbol “a-d” indicate the significant difference between seasons.

**Figure 1:**
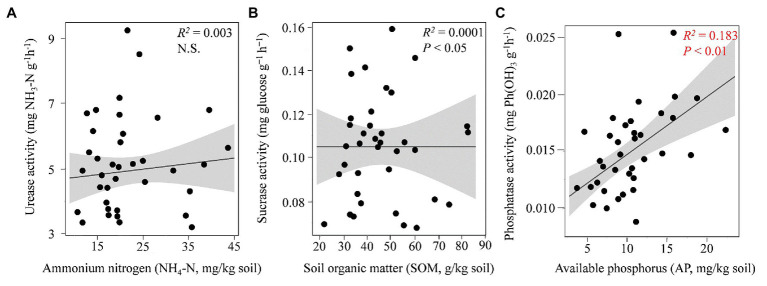
Relationship between edaphic properties and soil enzymatic activities. The relationships between ammonium-nitrogen (NH_4_-N) and urease **(A)**, soil organic matter (SOM) and sucrase **(B)**, and available phosphorus (AP) and phosphatase **(C)** are shown by the linear regression analysis. The red color indicates a significant correlation.

Among the tested soil enzymatic activities, the urease (*p* < 0.05), phosphatase (*p* < 0.001), and catalase (*p* < 0.001) activities were significantly affected by the changes in the seasons; however, the activities of sucrase and dehydrogenase did not differ significantly (*p* ≥ 0.05) among the four seasons (Table 1). The regression analysis indicated that the concentration of soil AP was significantly related to the phosphatase activity, while no obvious correlations were found between sucrose and SOM or between urease and N content (Figure 1, Supplementary Table S1). Spearman’s correlation analysis (Supplementary Table S1) revealed that the soil catalase activity was strongly positively correlated with the AP and NH_4_-N contents (*ρ* > 0.6; *p* < 0.01) but negatively correlated with the TP content.

### Root Fungal Colonization Rate

**Figure 2:**
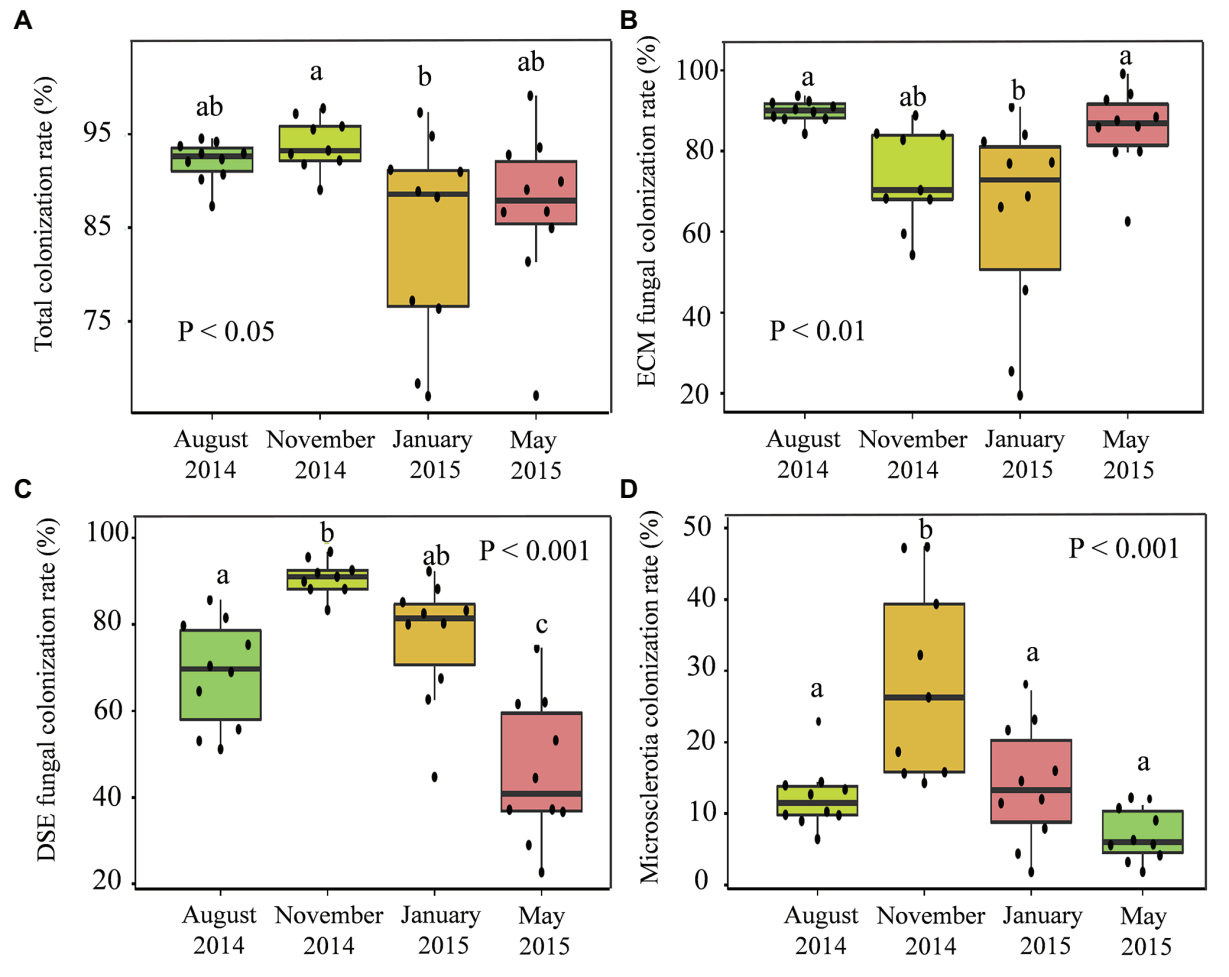
Seasonal changes in the root fungal colonization rates in temperate *Pinus tabuliformis* forests located on the Qinling Mountains. **(A)** Total fungal colonization rate; **(B)** ECM fungal colonization rate; **(C)** DSE fungal colonization rate; **(D)** microsclerotia colonization rate. ^∗^*p* < 0.05, ^∗∗^*p* < 0.01, ^∗∗∗^*p* < 0.001.

The fungal colonization rates reflected the colonization rates of the ECM fungi and DSEs that had colonized the *P. tabuliformis* roots (Figure 2). The total fungal (*p* < 0.05; Figure 2A), DSE hyphal (*p* < 0.001; Figure 2C), and microsclerotium (*p* < 0.001; Figure 2D) colonization rates increased from spring to summer, peaked in autumn (93.94, 90.81, and 28.53%, respectively) and then decreased in the winter, exhibiting a hump-shaped relationship. Likewise, the colonization rate of ECM fungi (Figure 2B) ranged from 63.68 to 89.77% across the seasons; moreover, the rate increased from spring and peaked in the summer, after which it significantly decreased from autumn to winter, with the lowest value occurring in winter (*p* < 0.01).

**Figure 3:**
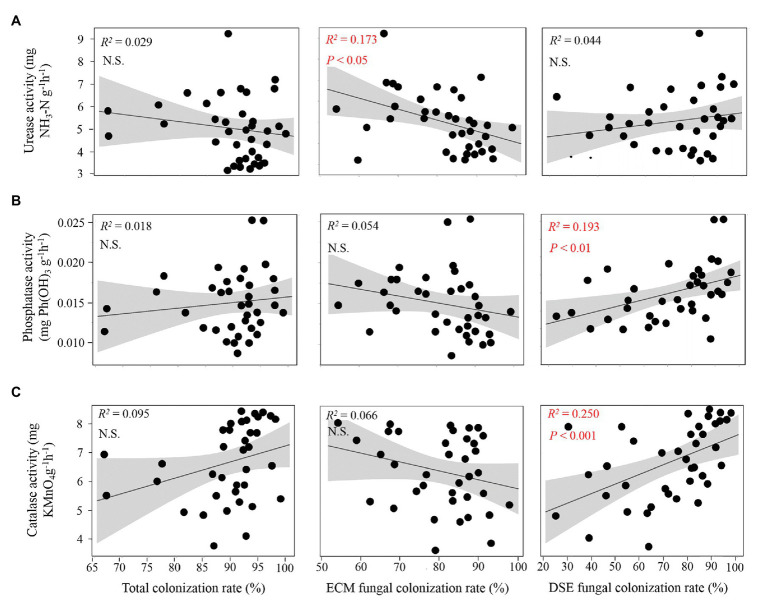
Relationship between soil enzymatic activities and root fungal colonization rates. **(A)** The relationships between urease activity and fungal colonization rate of overall fungi, ectomycorrhizal (ECM) fungi and dark septate endophyte (DSE) fungi; **(B,C)** the relationships between fungal colonization rates and the soil enzymatic activities of phosphatase and catalase. The red color indicates a significant correlation.

According to the correlation analysis, significant linear relationships were found between root fungal colonization rates and soil enzymatic activities (Figure 3). Additionally, a higher colonization rate of ECM fungi was found with a significant decline in soil urease (Figure 3A). Moreover, significantly increased soil phosphatase and catalase activities were exhibited with a greater colonization rate of DSE fungi in the present study (Figures 3B,C). The Spearman’s relationships showed that the total fungal colonization rate was strongly positively correlated with the SMC and ST (*ρ* > 0.7, *p* < 0.01; Supplementary Table S2). In addition, the ECM fungal colonization rate was negatively correlated with the AK content, while the DSE fungal colonization rate was significantly correlated with soil TK, AP, and NH_4_-N.

### Distribution of Fungal Species Across Seasons

A total of 568,821 raw reads were obtained from the 12 mixed soil and 12 mixed root amplicons, and this number decreased to 480,000 after normalization (20,000 per sample). Overall, 480,000 fungal sequences of 80 OTUs were recorded from the *P. tabuliformis* root tips and rhizospheric soils. The comparison of OTU abundance between samples showed that the fungal community composition and structure associated with the *P. tabuliformis* roots differed significantly from those in the *P. tabuliformis* rhizospheric soils (PERMANOVA; relative abundance: *pseudo-F* = 25.97, *R*^2^ = 0.541, *p* < 0.001; presence/absence: *pseudo-F* = 160.82, *R*^2^ = 0.880, *p* < 0.001). In addition, significant differences were also found in the composition of the fungal community associated with the roots (PERMANOVA; relative abundance: *pseudo-F* = 17.44, *R*^2^ = 0.867, *p* < 0.01; presence/absence: *pseudo-F* = 20.97, *R*^2^ = 0.887, *p* < 0.001) and in the rhizospheric soils across seasons (PERMANOVA; relative abundance: *pseudo-F* = 17.928, *R*^2^ = 0.871, *p* < 0.01; presence/absence: *pseudo-F* = 9.709, *R*^2^ = 0.785, *p* < 0.001).

**Figure 4:**
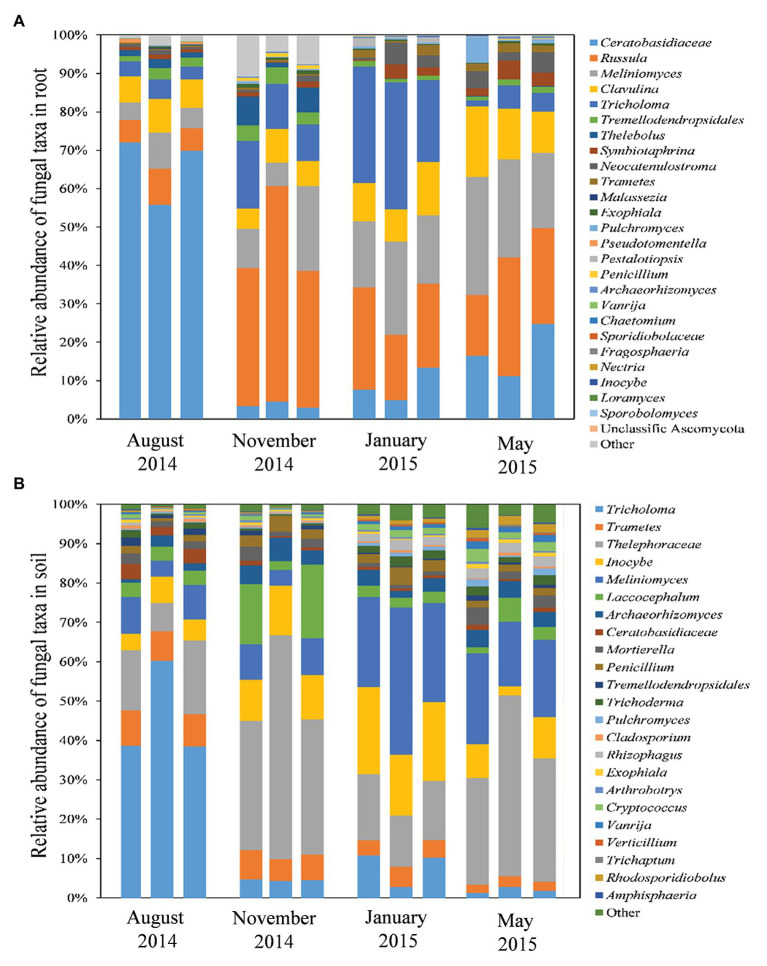
Composition and structure of fungal communities in roots **(A)** and rhizospheric soils **(B)** in *P. tabuliformis* forests across seasons.

The read numbers of species varied greatly between seasons for both the root and soil samples (Figure 4). Twenty-nine OTUs that belonged to the hyphae of Basidiomycota (73.5%), Ascomycota (24.12%), and other fungal lineages (2.37%) were detected in the *P. tabuliformis* root samples. The OTU identified as Ceratobasidiaceae was dominant in root samples collected during summer (65.9%) but significantly decreased in samples collected during spring (17.46%), winter (8.68%), and autumn (3.59%). However, the OTU identified as *Russula* has the highest number of reads in autumn (42.55%) and then declined in samples obtained during the spring (23.92%), winter (21.87%), and summer (7.03%). *Meliniomyces* shared similar properties of total reads in samples collected during the spring (25.1%) and winter (19.13%), significantly higher than those in the samples collected during autumn (12.6%) and summer (6.33%). *Tricholoma* was the predominant species in winter (28.24%) but showed lower abundance in the autumn (12.92%), spring (4.21%), and summer (4.08%). The number of OTUs present in the root samples differed significantly between the warm and cold seasons, with more OTUs present during the cold seasons. Among the 29 OTUs, 18 were found in all four seasons, 6 were found only in the cold seasons, and 2 were found only in the warm seasons (Supplementary Figure S1A). In addition, 13 OTUs were identified as belonging to fungi that form symbioses with *P. tabuliformis* roots (Figure 4A, Supplementary Table S3). *Tricholoma*, Ceratobasidiaceae, and *Meliniomyces variabilis* were detected mainly in cold seasons, while Ceratobasidiaceae and *Russula* were detected mainly in warm seasons.

With respect to the rhizospheric soil, 65 OTUs were detected, including Basidiomycota (68.58%), Ascomycota (27.03%), Mucoromycota (3.46%), Glomeromycota (0.05%), Chytridiomycota (0.04%), and Zoopagomycota (0.02%; Figure 4B). The *Tricholoma* was predominant in soil samples collected during the summer (45.38%) but had few reads in soils collected during the winter (7.88%), autumn (4.48%), and spring (1.85%). However, the taxa Thelephoraceae was dominant in soils collected both during the autumn (41.08%) and spring (34.14%), followed by the soils collected during the winter (14.94%) and summer (13.59%). *Meliniomyces* and *Inocybe* showed the highest number of reads in the soils collected during the winter (28.41 and 16.87%, respectively), and then, a decrease was observed in the soil samples collected during spring (19.36 and 6.57%, respectively), autumn (7.38 and 11.24%, respectively), and summer (7.36 and 5.08%, respectively). The relative abundance of OTUs in the rhizospheric soil samples also differed significantly between the warm and cold seasons, with more OTUs present during the latter. In total, 39 OTUs were detected in all four seasons; 2 OTUs occurred only during the warm seasons, and 10 OTUs were found only in the cold seasons (Supplementary Figure S1B).

### Temporal Changes in the Fungal Community

Although the dominant species was different between seasons, symbiotic fungi dominated the root-associated fungal communities. The symbiotic fungi shared most of the reads in both the root (90.16%) and rhizospheric soil (70.92%), and the soil had more shared reads belonging to the saprotrophic fungus than the root (Supplementary Figure S2A–D). The tracking of individual OTUs revealed distinct temporal dynamics within different lifestyles of fungi (Supplementary Figure S2E,F). Many of the symbiotic fungal OTUs displayed seasonal variability in both the roots and rhizospheric soil associated with *P. tabuliformis*; for instance, some were not present during the spring but were subsequently present at later time points, while a smaller proportion of saprotrophic fungi and phytopathogenic fungi exhibited this type of seasonal cyclic pattern.

**Figure 5:**
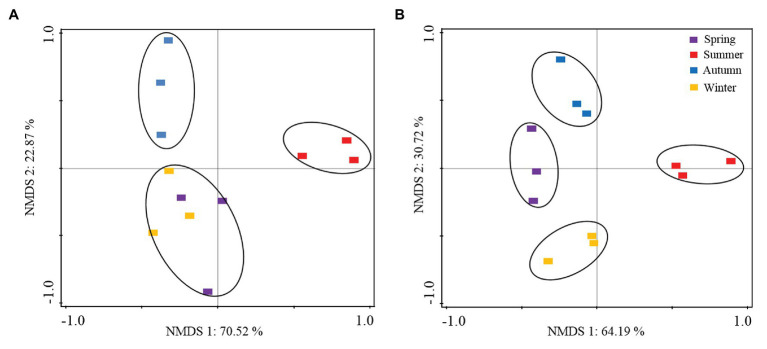
Nonmetric multidimensional scaling (NMDS) plots of the fungal community variation in *P. tabuliformis* forests based on the relative read abundance of operational taxonomic units (OTUs). **(A)** Root fungal community; **(B)** rhizospheric soil fungal community. Samples from seasons are indicated with distinct colors; fungal lifestyles in the community are indicated with distinct symbols.

The NMDS analysis revealed clear clustering of the relative abundance data associated with the *P. tabuliformis* root tips and in the rhizospheric soils during different seasons (Figure 5). The seasonal distribution of the samples and fungal species was depicted for both the root and rhizospheric soil fungal communities. Additionally, the principal groups of dominant species, corresponding to fungi present only during a specific period, were noted. For example, the dominant genera Ceratobasidiaceae and *Tricholoma* were mainly present in summer, while the genera *Russula* and Thelephoraceae predominated in autumn. In addition, the dominant genera *Meliniomyces*, *Tricholoma*, *Clavulina*, *Meliniomyces*, and *Inocybe* had high proportions in the fungal communities collected from winter and spring.

Spearman’s correlation analysis was conducted to determine the correlations between the abiotic factors and fungal community composition. The abundance of the dominant fungal phyla in the rhizospheric soil associated with *P. tabuliformis* was significantly correlated with the ST, SMC, and TP; moreover, in the root tip samples, only Basidiomycota was significantly negatively correlated with the pH (Supplementary Table S4). Moreover, Spearman’s relationships between abiotic factors and fungal taxa with total read abundances ≥0.1% of the total reads are shown in Supplementary Table S5. The edaphic properties of TP, ST, and SMC showed significant correlations with root-associated fungal taxa across seasons.

### Diversity Indicators of the Fungal Community

**Figure 6:**
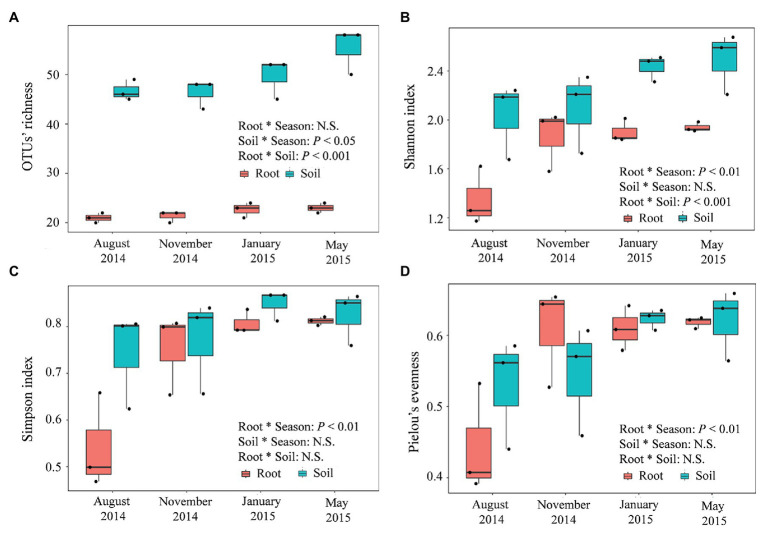
Seasonal changes in the alpha-diversities of the root-associated and rhizospheric soil fungal communities in *P. tabuliformis* forests located in the Qinling Mountains. **(A)** OTU richness, **(B)** Shannon, **(C)** Simpson, and **(D)** Pielou’s evenness. The horizontal bars within boxes represent the median. The tops and bottoms of boxes represent the 75th and 25th quartiles, respectively. The upper and lower whiskers extend 1.5× the interquartile range from the upper edge and lower edge of the box, respectively. N.S., non-significant.

Compared with the fungal communities in the roots of *P. tabuliformis*, those in the rhizospheric soil presented a greater alpha diversity, with greater OTU richness (Figure 6A), Shannon (Figure 6B), Simpson (Figure 6C), and Pielou’s evenness (evenness) index values (Figure 6D). However, the valley-shaped trend exhibited by these communities along with the seasonal changes was similar to that exhibited by the roots; the lowest alpha diversity values occurred in the summer and continuously increased from fall to winter, and the highest values occurred in the winter or spring. These results indicate that the richness and diversity of the fungal community decreased at higher temperatures. Additionally, decreased soil phosphatase and catalase activities were found to be related to greater soil fungal OTU richness (Supplementary Table S6), while only negative correlations were found between soil catalase activity and root fungal OTU richness. Moreover, the activity of soil urease increased as a consequence of the greater Simpson value of the soil and root fungal community.

### Networks and Connectedness

**Figure 7:**
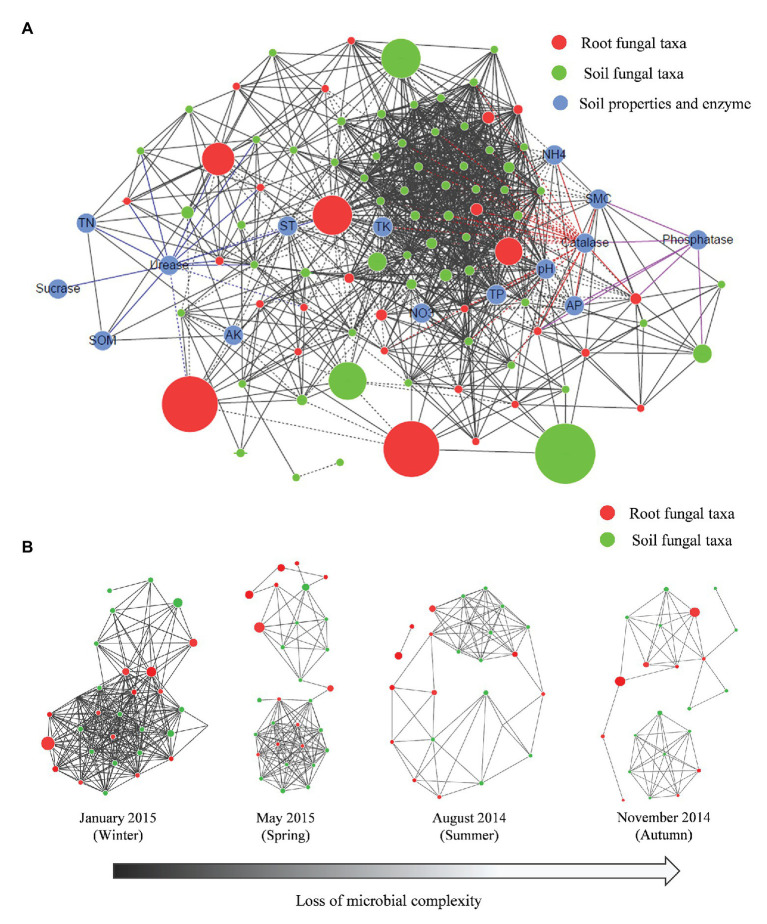
Network analysis showing the connectedness and complexity of fungal communities. **(A)** Network analysis between the fungal communities and edaphic factors in *P. tabuliformis* forests located in the Qinling Mountains; **(B)** network analysis showing the root-associated fungal connectedness and complexity along seasons across the year. A strong (Spearman’s *ρ* > 0.6) and significant (*p* < 0.01) linear relationship was observed. In the co-occurring network, colors indicate soil fungal taxa (green), root fungal taxa (red), and edaphic properties (blue); black lines indicate the significant connections between nodes, and colored lines indicate the fungal taxa that are significantly correlated with urease, catalase, and phosphatase. The sizes of nodes indicate the relative abundance of fungal taxa, solid lines indicate positive correlations, and dashed lines show negative correlations between nodes.

A network analysis was conducted to visualize the nonrandom co-occurrence and connectedness of the fungal taxa and edaphic properties. The network of root-associated fungal communities associated with *P. tabuliformis* comprised 103 nodes (88 OTUs and 15 edaphic properties) and 1,109 edges, with an average neighbor number of 20.835 and a characteristic path length of 2.153 (Figure 7A, Table 2). The network had an average clustering coefficient of 0.597 and an overall diameter of 6. More topological property values were also found in this network than in the Erdös-Rényi random network. The network analysis indicated that most fungal taxa were associated with TP, ST, and SMC, as well the soil enzymatic activities of urease and catalase. The network analysis of individual seasons illustrated that the low temperature in cold seasons had a strong effect on increasing the association network complexity of the root-associated fungal communities. Root-associated fungal network connectivity declined strongly in the summer and autumn (Figure 7B, Supplementary Table S7).

**Table 2:** Topological properties of co-occurring networks.

	Clustering coefficient (CC)	Average path length (APL)	Network diameter (ND)	Graph density (GD)	Average number of neighbors
Whole network	0.597	2.153	6	0.204	20.835
Random network	0.21	1.8	3	0.204	20.835

The random network was an identically sized random network generated based on the Erdős-Rényi model, whose topological five properties were calculated as the average value from 10,000 Erdős-Rényi random networks.

## Discussion

### Impact of Seasonal Changes on Edaphic Properties and Soil Enzymatic Activities

As described previously (Siles et al., 2016), climatic variables, such as air temperature, precipitation, and humidity, vary across seasons, directly leading to significant differences in ST and moisture. Seasonal changes in ST were found to be a consequence of the physical factors associated with altitude, precipitation, wind seasonality, site topography, soil water content, and soil texture, as well as surface litter and plant canopies (Paul et al., 2004; Körner, 2007). This finding explains why the ST recorded in the present study was strongly influenced by season but was always above the freezing point. Edaphic physicochemical properties have been linked to plant senescence, C input, and microorganism activity (Siles et al., 2016). The litter and SOM content in the *P. tabuliformis* forest soil increased with the reduced degradation rate that was caused by the temperature decline during the cold seasons, indicating that the SOM stocks were linked to temperature changes. The accumulation in SOM content increased the soil acidity because of the incomplete oxidation of plant litter (Wagai et al., 2011). The decomposition of plant litter depended on the abundance and composition of soil microorganisms and their activities, which were influenced by fluctuations in ST and SMC (Polyakova and Billor, 2007). Thus, an increase in the AP, AK, and NH_4_-N concentrations were detected in the warm seasons (Table 1).

The microbial enzymatic activities measured in the present study were significantly affected by seasonal changes. Contrasting results concerning variation in soil enzymatic activities in response to temperature fluctuations have occurred. Previous studies (Jing et al., 2014) have shown that, due to increased microbial biomass, the potential activities of soil extracellular enzymes are greater during the warm season than during the cold season. Thus, an increase in the soil catalase activity occurred during the warm seasons. However, the urease activity increased in response to declining temperatures because microbes can increase their production of enzymes to compensate for the relatively low enzyme reaction kinetics during the cold season (Bell et al., 2010). In addition to temperature, substrate availability and quality have also been reported to be key factors that control extracellular enzyme production (German et al., 2011). The sharp increase in AP absorption during warm seasons results in an increased phosphatase pool size (Figure 1).

### Effects of Seasonal Changes on the Diversity and Composition of Fungal Communities

Both the rhizospheric soil communities and root fungal communities of temperate *P. tabuliformis* forests were found to be strongly affected by seasonal changes. In this study, the seasonal changes in environmental factors significantly affected the structure of the rhizospheric soil and root fungal communities in *P. tabuliformis* forests according to the two-way PERMANOVA of the fungal community compositions. Moreover, significant differences between the root fungal communities and rhizospheric soil fungal communities were revealed. The distributional patterns of the fungal communities suggest that (i) the sensitivity and preference to temperature variation are different between these predominant species in fungal communities; (ii) only a small proportion of root fungi can produce extrametrical mycelia that extend into the surrounding soil, and the relative abundance of those fungi is very low in the rhizospheric soil fungal communities; and (iii) host plants exert strong selective pressure on their associated root microbiome, and the symbiotic fungal taxa have a strong tendency to be tree-specific (Urbanová et al., 2015).

The root-associated fungal communities of *P. tabuliformis* differed significantly among the four seasons, especially between the warm and cold seasons (Figure 4). Moreover, the dynamic changes between Basidiomycota and Ascomycota in response to the seasons were the opposite. This result suggested that members of Basidiomycota (such as ECM fungal taxa and ECM colonization rate) present a stronger competitive ability and colonization intensity at optimal temperatures, while those of Ascomycota (such as DSE fungal taxa and DSE colonization rate) have some selective advantages in terms of surviving under extremely cold conditions (Redman et al., 2002; Ban et al., 2012). Furthermore, more of the present new species occurred during the warm seasons than during the cold seasons in both the soil and root samples, while more unique species were found in the cold season. These findings indicate that the decrease in temperature and soil humidity leads to an increase in fungal richness. Additionally, the cluster of fungal community composition structure showed little difference between sampling sites in our study.

### Seasonal Changes in Root-Associated Fungal Microbiota Drive N and P Cycling

In the present study, soil microclimate factors and physicochemical properties, both of which are directly influenced by seasonal changes, strongly affected the underground fungal communities in *P. tabuliformis* forests in the Qinling Mountains. According to the correlation analysis, ST, SMC, and P in the soil presented the majority of associations with the OTUs in the *P. tabuliformis* root-associated fungal community (Figure 7). By regulating both fungal growth and the proportions of different microbial taxa, the seasonal changes in the edaphic properties led to differences in soil ecosystem functions among different seasons. The soil functional processes, especially N and P cycling, were found to be strongly associated with the fungal community compositions, which differ between seasons.

Soil ecosystems are highly heterogeneous as soil microbial communities are easily influenced by abiotic factors, and the spatial and temporal changes in microclimate induce fluctuating microbial biodiversity within soil aggregates (Rillig et al., 2017; Bach et al., 2018). The temporal heterogeneity of microbes along seasons determines the soil ecosystem functioning, which plays an important role in the nutrient cycling processes. The soil ecosystem functions are likely driven by not only the microbial abundance and richness but also the interactions between functional taxa. Considering the additional temporal variations in the soil indictors (edaphic properties and enzymatic activities) across the investigated seasons together with the fungal microbiota could suggest the correlations within functional taxa and terrestrial soil ecosystem. Moreover, the present study indicates that the seasonal changes in a temperate forest root-associated fungal microbiota induced the soil ecosystem fluctuation, especially the N and P cycling. The alternation of the functional microbial taxa along seasons result in the seasonal changes of nutrient cycling processes. Moreover, recent studies have suggested that the microbial network complexity varies between different ecosystem types (Kohlmeier et al., 2005). The network complexity of soil microbes was different at both horizontal and vertical scales, where the microbial communities with higher microbial diversity and functional guilds have higher network complexity (Wagg et al., 2019). Our work further illustrates that the microbial network complexity varies by season, which is responsible for the variations in soil functions. The rapid increase in the relative abundance of predominant taxa at higher temperature and soil humidity during warm seasons seems could lead to a decline of microbial network complexity. In addition, the soil cycling processes, such as N and P cycling, were significantly impacted by the seasonal changes in the microbial community. Thus, our work also demonstrates that global warming will not only lead to a decline in the richness of underground microbiota but also a loss of the microbial network complexity and further simplifies the functions of the soil ecosystem in the short term.

## Data Availability Statement

The original contributions presented in the study are publicly available. This data can be found here: https://www.ncbi.nlm.nih.gov/bioproject/PRJNA680752.

## Author Contributions

C-YW and H-HW designed and supervised this study. MT and S-XZ supervised this study. H-HW, H-LC, QD and HF performed the experiments. H-HW refined the data analysis and wrote the manuscript. All authors contributed to the article and approved the submitted version.

### Conflict of Interest

The authors declare that the research was conducted in the absence of any commercial or financial relationships that could be construed as a potential conflict of interest.
