# Supplementary material for: Seasonal Changes in Pinus tabuliformis Root-Associated Fungal Microbiota Drive N and P Cycling in Terrestrial Ecosystem
Source: Front Microbiol. 2021 Jan 18;11:526898. doi: 10.3389/fmicb.2020.526898 (PMC7849022; doi:10.3389/fmicb.2020.526898)

Supplementary Table S1. Spearman’s correlation coefficients between the rhizospheric soil enzymatic activities and edaphic factors in *Pinus tabulaeformis* forests.

|  | Sucrase | Urease | Dehydrogenase | Phosphatase | Catalase |
| --- | --- | --- | --- | --- | --- |
| SMC | 0.153846 | -0.27972 | 0.244755 | 0.517483 | 0.643* |
| ST | -0.17895 | -0.684* | 0.336844 | 0.017544 | 0.403511 |
| pH | -0.18881 | -0.21678 | 0.293706 | -0.26573 | -0.11189 |
| TK | -0.27273 | -0.55944 | 0.391608 | -0.587* | -0.48951 |
| TN | 0.622* | 0.650* | -0.22378 | 0.41958 | -0.02098 |
| TP | -0.02797 | 0.132867 | 0.048951 | -0.580* | -0.839** |
| AP | 0.265734 | 0.405594 | -0.42657 | 0.867** | 0.811** |
| AK | 0.111888 | 0.461538 | -0.37063 | 0.377622 | 0.160839 |
| NO_3_ -N | 0.013986 | -0.31469 | 0.706* | -0.34266 | -0.28671 |
| NH_4_-N | -0.01399 | -0.06294 | 0.104895 | 0.51049 | 0.797** |
| SOM | 0.433566 | 0.573427 | -0.24476 | 0.335664 | 0.167832 |

Data in red indicated significant correlations, *: significant at *P* < 0.05; **: significant at *P* < 0.01; ***: significant at *P* < 0.001.

Supplementary Table S2. Spearman’s correlation coefficients between the root fungal colonization rates and edaphic factors in *Pinus tabulaeformis* forests.

|  | Total fungal colonization rate | ECM fungal colonization rate | DSE colonization rate | Microsclerotia colonization rate |
| --- | --- | --- | --- | --- |
| SMC | 0.832** | 0.146853 | 0.454545 | 0.384615 |
| ST | 0.758** | 0.582* | 0.070176 | 0.112281 |
| pH | -0.02098 | 0.153846 | -0.35664 | -0.32168 |
| TK | 0.020979 | 0.643* | -0.748** | -0.56643 |
| TN | -0.33566 | -0.55944 | 0.412587 | 0.216783 |
| TP | -0.671* | 0.041958 | -0.657* | -0.48951 |
| AP | 0.244755 | -0.678* | 0.944** | 0.741** |
| AK | -0.43357 | -0.839** | 0.503497 | 0.384615 |
| NO_3_ -N | 0.314685 | 0.503497 | -0.41958 | -0.34965 |
| NH_4_-N | 0.51049 | -0.1958 | 0.727** | 0.391608 |
| SOM | -0.33566 | -0.587* | 0.517483 | 0.370629 |

Data in red indicated significant correlations, *: significant at *P* < 0.05; **: significant at *P* < 0.01; ***: significant at *P* < 0.001.

Supplementary Table S3 Mycorrhizal fungi recorded in roots and soils of *Pinus tabulaeformis* at different seasons, based on the NCBI blast searches.

|  | Rhizospheric soil | |  |  | Root | |  |  |  |
| --- | --- | --- | --- | --- | --- | --- | --- | --- | --- |
| Taxa | Summer  (August 2014) | Autumn  (November 2014) | Winter  (January 2015) | Spring  (May 2015) | Summer  (August 2014) | Autumn  (November 2014) | Winter  (January 2015) | Spring  (May 2015) | Accession number |
| Thelephorales | 13.593±3.347a | 41.083±7.654b | 14.943±1.117a | 34.143±5.71b |  |  |  |  | UDB028526 |
| Tricholoma | 45.378±7.193a | 4.477±0.133b | 7.883±2.608b | 1.852±0.446b | 4.075±0.486c | 12.952±2.411b | 28.237±3.563a | 4.208±1.395c | UDB028531 |
| Meliniomyces | 7.363±1.694c | 7.38±1.706c | 28.41±4.556a | 19.355±1.839b |  |  |  |  | AY838785.1 |
| Inocybaceae | 5.075±0.759c | 11.24±0.638b | 16.873±1.029a | 6.567±2.484c | 0 | 0 | 0.038±0.022a | 0 | UDB028517 |
| Rhizophagus | 0.473±0.141b | 0.317±0.065b | 2.225±0.188a | 2.48±0.028a |  |  |  |  | FJ009617.1 |
| Ceratobasidiaceae | 3.205±0.614a | 0.723±0.24b | 0.74±0.081b | 1±0.248b | 65.897±5.074a | 3.59±0.506c | 8.678±2.496bc | 17.462±3.973b | UDB028535 |
| Tremellodendropsidales | 1.413±0.381a | 0.733±0.318ab | 0.297±0.043b | 0.823±0.222ab | 2.205±0.493b | 3.808±0.354a | 1.177±0.163b | 1.357±0.218b | UDB028538 |
| Inocybe (Fr.) Fr. | 0.293±0.032b | 0.147±0.02b | 2.33±0.929a | 0.477±0.095b |  |  |  |  | [UDB013456](https://unite.ut.ee/bl_forw.php?id=343690) |
| Diversispora | 0 | 0.017±0.008b | 0.012±0.002b | 0.138±0.066a |  |  |  |  | AM713425.1 |
| Dentiscutata | 0.005±0.003b | 0 | 0 | 0.038±0.007a |  |  |  |  | AJ871271.1 |
| Russulaceae | 0 | 0 | 0.003±0.003a | 0 |  |  |  |  | FM993249 |
| Lactarius | 0 | 0.003±0.003ab | 0.027±0.015a | 0.003±0.003ab |  |  |  |  | HQ328776.1 |
| Inocybe australiensis | 0.113±0.066a | 0.01±0.006a | 0 | 0 |  |  |  |  | KJ756468.1 |
| Russula |  |  |  |  | 7.033±1.171a | 42.553±6.782c | 21.87±2.8b | 23.917±4.385b | AY293156.1 |
| Meliniomyces variabilis |  |  |  |  | 6.332±1.428c | 12.603±4.81bc | 19.127±2.17ac | 25.095±3.304a | FJ430779.1 |
| Clavulina |  |  |  |  | 7.678±0.593b | 6.858±0.995b | 10.748±1.637ab | 14.068±2.276a | AY757265.1 |
| Meliniomyces |  |  |  |  | 0.077±0.045b | 0.227±0.124b | 0.578±0.115a | 0.208±0.08b | AY838789.1 |
| Pseudotomentella |  |  |  |  | 0.518±0.247a | 0 | 0.003±0.003b | 0 | UDB028693 |
| Unclassific Ascomycota |  |  |  |  | 0 | 0 | 0.023±0.015a | 0 | AH008235.2 |

The data represented the presence/absence and relative abundance (%) of taxa. Blank represented the absence of taxa in root-associated and rhizospheric soil fungal communities of *P*. *tabulaeformis*; the data were calculated based on number of reads.

Supplementary Table S4. Spearman’s correlation coefficients between the edaphic factors and relative abundant of phyla in root and soil fungal communities in *Pinus tabulaeformis* forests.

|  | Root |  | Rhizospheric soil |  |  |  |  |  |
| --- | --- | --- | --- | --- | --- | --- | --- | --- |
|  | Basidiomycota | Ascomycota | Ascomycota | Basidiomycota | Chytridiomycota | Glomeromycota | Kickxellomycotina | Mucoromycotina |
| SMC | 0.098 | -0.329 | -0.685* | 0.685* | 0.021 | -0.141 | -0.482 | -0.273 |
| ST | 0.428 | -0.554 | -0.874** | 0.842** | -0.257 | -0.356 | -0.42 | -0.449 |
| pH | -0.594* | 0.566 | 0.133 | -0.161 | 0.780** | 0.763** | 0.241 | 0.573 |
| TK | -0.21 | 0.252 | 0.007 | -0.098 | 0.418 | 0.541 | 0.277 | 0.601* |
| TN | 0.063 | 0 | 0.413 | -0.399 | -0.197 | -0.279 | 0.248 | 0.042 |
| TP | -0.287 | 0.49 | 0.671* | -0.713** | 0.46 | 0.474 | 0.688* | 0.685* |
| AP | -0.049 | -0.133 | -0.133 | 0.224 | -0.285 | -0.336 | -0.575 | -0.469 |
| AK | -0.329 | 0.371 | 0.455 | -0.378 | 0.028 | -0.025 | 0.021 | -0.098 |
| NO_3_-N | -0.119 | 0.133 | -0.189 | 0.112 | 0.587* | 0.41 | 0.284 | 0.448 |
| NH_4_-N | 0.112 | -0.245 | -0.538 | 0.573 | -0.38 | -0.378 | -0.688* | -0.538 |
| SOM | 0.077 | -0.007 | 0.329 | -0.301 | -0.271 | -0.357 | 0.092 | -0.105 |

Data in red indicate significant correlations, *: significant at *P* < 0.05; **: significant at *P* < 0.01; ***: significant at *P* < 0.001.

Supplementary Table S5. Spearman’s correlation coefficients between the edaphic factors and relative abundant of fungal taxa in root and soil fungal communities in *Pinus tabulaeformis* forests.

|  | SMC | ST | pH | TK | TN | TP | AP | AK | NO3 | NH4 | SOM |
| --- | --- | --- | --- | --- | --- | --- | --- | --- | --- | --- | --- |
| Root fungal taxa |  |  |  |  |  |  |  |  |  |  |  |
| *Ceratobasidiaceae* | -0.238 | 0.330 | -0.084 | 0.545 | -0.294 | 0.217 | -0.762** | -0.706* | 0.168 | -0.441 | -0.357 |
| *Russula* | 0.252 | -0.274 | 0.252 | -0.413 | 0.231 | -0.056 | 0.531 | 0.594* | 0.175 | 0.357 | 0.308 |
| *Meliniomyces* | -0.385 | -0.572 | 0.580* | 0.259 | 0.007 | 0.559 | -0.196 | 0.371 | 0.168 | -0.287 | 0.007 |
| *Tricholoma* | -0.287 | -0.575 | -0.427 | -0.839** | 0.559 | -0.098 | 0.678* | 0.713** | -0.608* | 0.308 | 0.678* |
| *Clavulina* | -0.713** | -0.505 | 0.378 | 0.238 | 0.140 | 0.748** | -0.657* | 0.182 | 0.294 | -0.490 | 0.189 |
| *Teratosphaeriaceae* | -0.445 | -0.401 | 0.469 | 0.420 | -0.158 | 0.483 | -0.368 | 0.053 | 0.053 | -0.354 | -0.140 |
| *Tremellodendropsidales* | 0.734** | 0.558 | 0.091 | -0.147 | -0.357 | -0.601* | 0.378 | -0.133 | 0.245 | 0.517 | -0.287 |
| *Symbiotaphrina* | -0.427 | -0.354 | 0.538 | 0.420 | -0.224 | 0.545 | -0.364 | 0.070 | 0.161 | -0.280 | -0.098 |
| *Thelebolus* | 0.886** | 0.777** | 0.084 | 0.116 | -0.452 | -0.827** | 0.375 | -0.483 | 0.063 | 0.518 | -0.466 |
| *Trametes* | -0.669* | -0.772** | 0.403 | -0.063 | 0.326 | 0.746** | -0.235 | 0.585* | 0.147 | -0.368 | 0.441 |
| *Ascodesmidaceae* | -0.385 | -0.670* | 0.364 | 0.077 | 0.147 | 0.392 | -0.147 | 0.182 | 0.028 | -0.441 | -0.014 |
| *Exophiala* | 0.280 | -0.186 | 0.350 | -0.168 | -0.077 | -0.091 | 0.399 | 0.280 | 0.175 | 0.259 | -0.056 |
| *Pestalotiopsis* | -0.694* | -0.838** | -0.035 | -0.263 | 0.294 | 0.469 | 0.035 | 0.361 | -0.385 | -0.427 | 0.308 |
| *Meliniomyces* | -0.448 | -0.707* | -0.140 | -0.382 | 0.235 | 0.123 | 0.298 | 0.375 | -0.536 | -0.189 | 0.196 |
| *Eurotiales* | 0.741** | 0.568 | 0.161 | -0.098 | -0.350 | -0.538 | 0.427 | -0.007 | 0.273 | 0.650* | -0.196 |
| *Archaeorhizomyces* | -0.070 | -0.418 | 0.606* | 0.179 | 0.147 | 0.557 | -0.161 | 0.417 | 0.595* | -0.182 | 0.095 |
| *Pseudotomentella* | 0.083 | 0.514 | -0.574 | 0.046 | -0.141 | -0.316 | -0.179 | -0.628* | -0.241 | -0.037 | -0.233 |
| Soil fungal taxa |  |  |  |  |  |  |  |  |  |  |  |
| *Rhizophagus* | -0.769** | -0.702* | 0.105 | 0.273 | 0.294 | 0.846** | -0.531 | 0.287 | 0.042 | -0.650* | 0.161 |
| *Thelephoraceae* | 0.455 | 0.060 | 0.490 | 0.126 | -0.049 | 0.112 | 0.133 | 0.259 | 0.608* | 0.119 | -0.056 |
| *Meliniomyces* | -0.804** | -0.842** | -0.028 | -0.070 | 0.434 | 0.657* | -0.161 | 0.385 | -0.343 | -0.538 | 0.385 |
| *Tricholoma* | 0.189 | 0.544 | -0.552 | -0.308 | -0.154 | -0.573 | 0.203 | -0.448 | -0.413 | 0.294 | -0.014 |
| *Inocybaceae* | -0.350 | -0.663* | -0.371 | -0.804** | 0.580* | -0.063 | 0.573 | 0.741** | -0.636* | 0.070 | 0.580* |
| *Polyporaceae* | 0.497 | 0.533 | 0.182 | 0.147 | -0.636* | -0.399 | 0.210 | -0.469 | 0.042 | 0.259 | -0.476 |
| *Trametes* | 0.587* | 0.761** | -0.497 | -0.189 | -0.245 | -0.790** | 0.406 | -0.420 | -0.315 | 0.503 | -0.161 |
| *Eurotiales* | 0.035 | -0.439 | -0.273 | -0.699* | 0.657* | -0.133 | 0.769** | 0.783** | -0.364 | 0.406 | 0.720** |
| *Archaeorhizomyces* | -0.601* | -0.677* | 0.252 | -0.091 | 0.336 | 0.664* | -0.301 | 0.378 | 0.231 | -0.371 | 0.378 |
| *Mortierellaceae* | 0.524 | 0.382 | 0.664* | 0.741** | -0.441 | 0.014 | -0.315 | -0.580* | 0.664* | -0.189 | -0.587* |
| *Hypocreales* | -0.680* | -0.645* | -0.018 | 0.256 | 0.270 | 0.676* | -0.417 | 0.102 | -0.151 | -0.718** | 0.091 |
| *Ceratobasidiaceae* | 0.182 | 0.491 | -0.098 | 0.462 | -0.378 | -0.210 | -0.378 | -0.860** | 0.028 | -0.273 | -0.524 |
| *Cryptococcus* | -0.602* | -0.756** | 0.392 | 0.347 | 0.249 | 0.816** | -0.420 | 0.228 | 0.172 | -0.655* | 0.116 |
| *Archaeorhizomyces* | 0.594* | 0.186 | 0.559 | 0.105 | -0.217 | -0.210 | 0.196 | 0.126 | 0.552 | 0.294 | -0.238 |
| *Sporidiobolaceae* | -0.671* | -0.649* | 0.350 | 0.448 | 0.147 | 0.951** | -0.601* | 0.133 | 0.273 | -0.727** | 0.070 |
| *Tremellodendropsidales Vizzini* | 0.434 | 0.551 | 0.231 | 0.657* | -0.503 | -0.126 | -0.301 | -0.783** | 0.280 | -0.266 | -0.643* |
| *Inocybaceae* | -0.902** | -0.702* | -0.168 | -0.049 | 0.350 | 0.734** | -0.378 | 0.168 | -0.252 | -0.601* | 0.364 |
| *Vanrija* | -0.678* | -0.653* | 0.350 | 0.441 | 0.189 | 0.923** | -0.615* | 0.189 | 0.238 | -0.741** | 0.063 |
| *Ascodesmidaceae* | -0.653* | -0.634* | 0.189 | 0.274 | 0.084 | 0.554 | -0.474 | -0.007 | -0.046 | -0.639* | -0.067 |
| *Exophiala* | -0.049 | -0.225 | 0.629* | 0.608* | 0.007 | 0.608* | -0.476 | -0.217 | 0.629* | -0.517 | -0.161 |
| *Cladosporium cladosporioides* | -0.294 | -0.025 | -0.056 | 0.469 | -0.098 | 0.490 | -0.469 | -0.336 | 0.105 | -0.483 | -0.098 |
| *Pleomassariaceae* | -0.680* | -0.835** | 0.200 | 0.018 | 0.504 | 0.739** | -0.217 | 0.595* | -0.004 | -0.420 | 0.441 |
| *Arthrobotrys* | -0.042 | -0.285 | 0.039 | 0.126 | 0.287 | 0.130 | 0.081 | -0.102 | -0.112 | -0.249 | 0.140 |
| *Teratosphaeria* | -0.581* | -0.735** | 0.004 | 0.025 | 0.406 | 0.525 | -0.140 | 0.270 | -0.252 | -0.609* | 0.256 |
| *Fragosphaeria* | -0.648* | -0.880** | 0.242 | -0.011 | 0.501 | 0.711** | -0.179 | 0.606* | 0.007 | -0.452 | 0.413 |
| *Taiwanofungus* | 0.081 | -0.148 | 0.744** | 0.488 | -0.316 | 0.263 | -0.246 | -0.260 | 0.432 | -0.330 | -0.393 |
| *Verticillium* | -0.792** | -0.664* | 0.172 | 0.382 | 0.154 | 0.802** | -0.644* | 0.032 | 0.021 | -0.792** | 0.004 |
| *Amphisphaeriaceae* | -0.531 | -0.463 | 0.049 | 0.266 | 0.126 | 0.503 | -0.448 | -0.133 | -0.126 | -0.783** | -0.098 |
| *Cryptotrichosporon* | -0.633* | -0.697* | 0.429 | 0.373 | 0.193 | 0.931** | -0.527 | 0.151 | 0.313 | -0.689* | 0.130 |
| *Acidea* | -0.330 | -0.579* | 0.540 | 0.460 | 0.060 | 0.646* | -0.351 | 0.028 | 0.309 | -0.558 | -0.077 |
| *Trichaptum* | 0.203 | -0.049 | 0.711** | 0.417 | -0.434 | 0.116 | 0.021 | -0.109 | 0.287 | -0.049 | -0.354 |
| *Cryptococcus* | -0.615* | -0.793** | 0.245 | 0.014 | 0.476 | 0.699* | -0.182 | 0.636* | 0.035 | -0.357 | 0.434 |
| *Symbiotaphrina* | -0.684* | -0.822** | 0.221 | 0.102 | 0.498 | 0.789** | -0.361 | 0.368 | 0.074 | -0.607* | 0.326 |

Data in red indicate significant correlations, *: significant at *P* < 0.05; **: significant at *P* < 0.01; ***: significant at *P* < 0.001.

Supplementary Table S6. Spearman’s correlation coefficients between the soil enzymatic activities and fungal diversity indexes in root and soil of *Pinus tabulaeformis* forests.

|  |  | Root |  |  |  | Soil |  |  |  |
| --- | --- | --- | --- | --- | --- | --- | --- | --- | --- |
|  |  | Simpson | OTUs richness | Evenness | Shannon | Simpson | OTUs richness | Evenness | Shannon |
|  | Sucrase | -0.15385 | 0.079171 | -0.1958 | -0.13986 | 0.48951 | -0.01058 | 0.398601 | 0.426573 |
|  | Urease | 0.384615 | 0.297836 | 0.363636 | 0.328671 | 0.685* | 0.095242 | 0.559441 | 0.587* |
|  | Dehydrogenase | -0.09091 | -0.12441 | -0.0979 | -0.03497 | -0.51748 | -0.01764 | -0.38462 | -0.27273 |
|  | Phosphatase | -0.37063 | -0.19604 | -0.29371 | -0.27273 | 0.20979 | -0.49385 | 0.027972 | 0.013986 |
|  | Catalase | -0.39161 | -0.48257 | -0.23776 | -0.20979 | -0.23776 | -0.698* | -0.33566 | -0.37063 |
| Root | Simpson | 1 | 0.52781 | 0.972** | 0.944** | 0.622* | 0.667* | 0.699* | 0.727** |
|  | OTUs richness | 0.52781 | 1 | 0.426018 | 0.55043 | 0.656* | 0.879** | 0.471259 | 0.54289 |
|  | Evenness | 0.972** | 0.426018 | 1 | 0.958** | 0.580* | 0.550286 | 0.692* | 0.713** |
|  | Shannon | 0.944** | 0.55043 | 0.958** | 1 | 0.580* | 0.638* | 0.629* | 0.685* |
| Soil | Simpson | 0.622* | 0.656* | 0.580* | 0.580* | 1 | 0.614* | 0.916** | 0.909** |
|  | OTUs richness | 0.667* | 0.879** | 0.550286 | 0.638* | 0.614* | 1 | 0.586* | 0.642* |
|  | Evenness | 0.699* | 0.471259 | 0.692* | 0.629* | 0.916** | 0.586* | 1 | 0.979** |
|  | Shannon | 0.727** | 0.54289 | 0.713** | 0.685* | 0.909** | 0.642* | 0.979** | 1 |

Data in red indicate significant correlations, *: significant at *P* < 0.05; **: significant at *P* < 0.01; ***: significant at *P* < 0.001.

Supplementary **Table S7** Topological properties of co-occurring networks.

|  | Clustering coefficient (CC) | Average path length (APL) | Network diameter (ND) | Graph density (GD) | Average number of neighbors | Network centralization |
| --- | --- | --- | --- | --- | --- | --- |
| August 2014 (Summer) | 0.682 | 2.323 | 5 | 0.29 | 6.667 | 0.158 |
| November 2014 (Autumn) | 0.696 | 2.168 | 7 | 0.238 | 5.0 | 0.105 |
| January 2015 (Winter) | 0.815 | 1.553 | 5 | 0.553 | 16.581 | 0.229 |
| May 2015 (Spring) | 0.687 | 3.192 | 8 | 0.323 | 9.034 | 0.190 |

Supplementary Figure S1. Venn-diagrams of fungal communities in *Pinus tabuliformis* forests based on the numbers of OTUs presence/absence in the fungal communities from different seasons. (A) Root fungal community; (B) rhizospheric soil fungal community


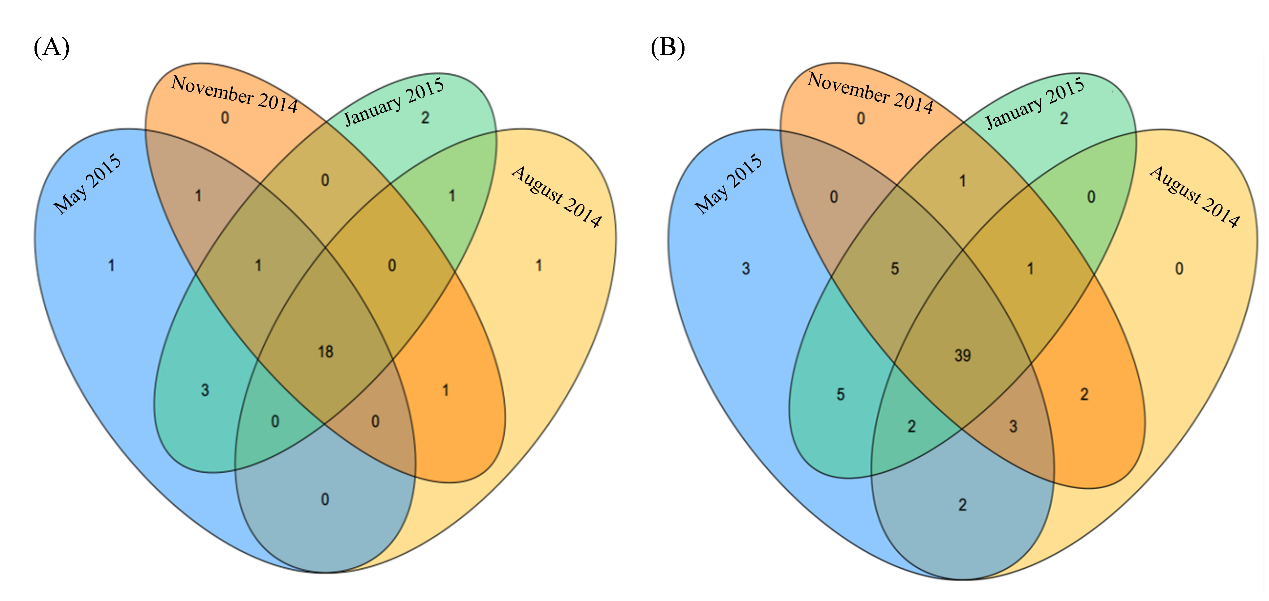


Supplementary Figure S2. Fungal community compositions in *Pinus tabuliformis* forests were cycling and could be differentiated by season. The read numbers of different lifestyles of fungi that were presented in the indicated seasons in roots (A) and rhizospheric soils (B) are colored by lifestyle on a stream graph. The OTU numbers of different lifestyles of fungi that were present in different seasons in roots (C) and rhizospheric soils (D) are colored by lifestyle on a stream graph. OTUs of lifestyles that were presented within each season were tracked by using Sankey plots in both the roots (E) and rhizospheric soils (F). The height of the rectangles indicates the number of OTUs; the lines represent the transfer of OTUs among different seasons; different lifestyles of fungi are indicated by distinct colors; the word “New” in the figure represents OTUs that were absent in the previous season.


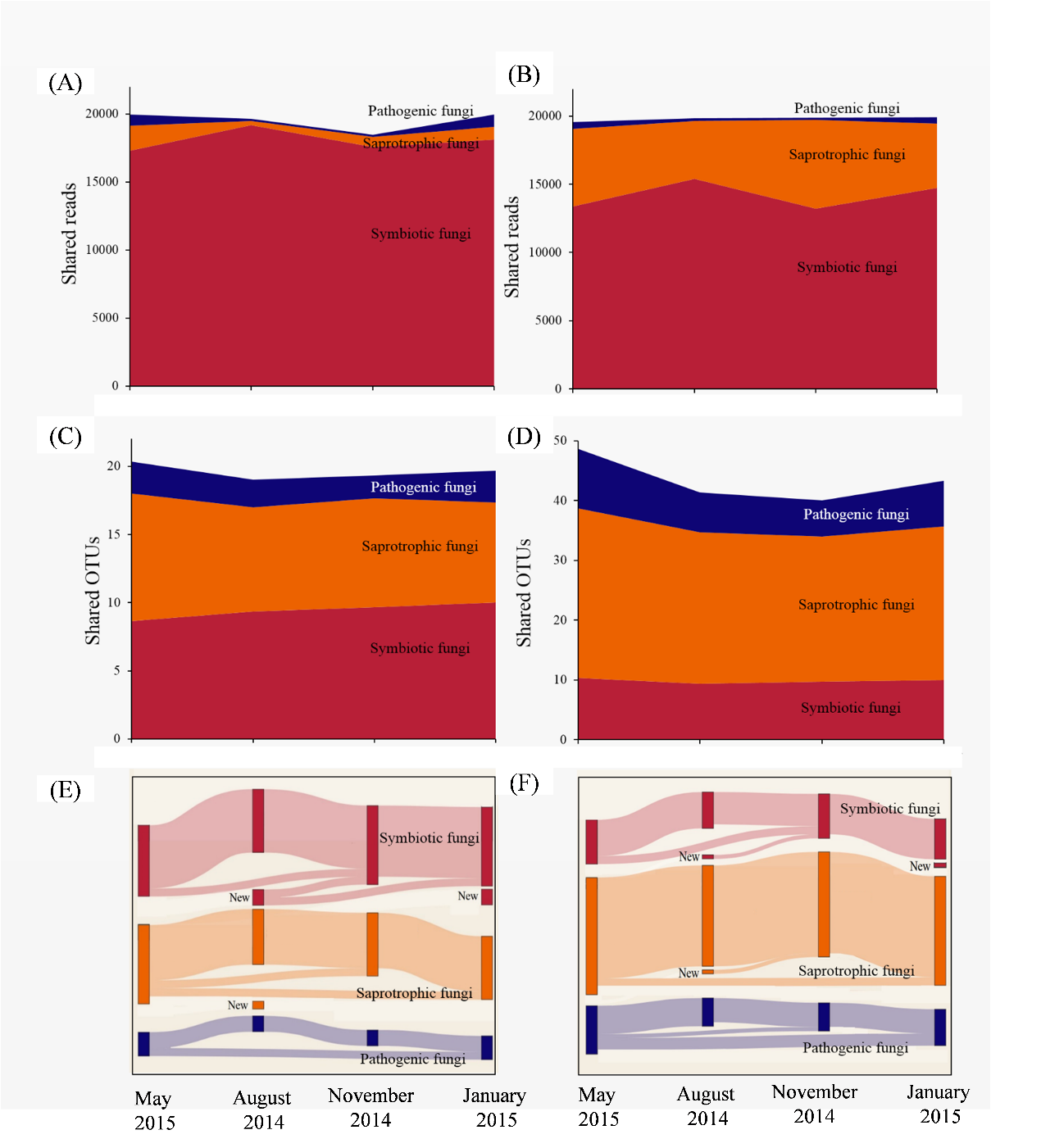

Supplement: Supplementary file 1 [file Data_Sheet_1.docx]
